# Acute effects of ventilator hyperinflation with increased inspiratory time on respiratory mechanics: randomized crossover clinical trial

**DOI:** 10.5935/0103-507X.20190052

**Published:** 2019

**Authors:** Luciano Matos Chicayban

**Affiliations:** 1 Laboratório de Pesquisa em Fisioterapia Pneumofuncional e Intensiva, Institutos Superiores de Ensino do CENSA - Campos dos Goytacazes (RJ), Brasil.; 2 Unidade de Terapia Intensiva, Hospital Geral de Guarus - Campos dos Goytacazes (RJ), Brasil.

**Keywords:** Physical therapy modalities, Respiration, artificial, Respiratory therapy/methods, Respiratory care units, Respiratory mechanics, Positive-pressure respiration

## Abstract

**Objective:**

To evaluate the effects of ventilator hyperinflation on respiratory mechanics.

**Methods:**

A randomized crossover clinical trial was conducted with 38 mechanically ventilated patients with pulmonary infection. The order of the hyperinflation and control (without changes in the parameters) conditions was randomized. Hyperinflation was performed for 5 minutes in pressure-controlled ventilation mode, with progressive increases of 5cmH_2_O until a maximum pressure of 35cmH_2_O was reached, maintaining positive end expiratory pressure. After 35cmH_2_O was reached, the inspiratory time and respiratory rate were adjusted so that the inspiratory and expiratory flows reached baseline levels. Measurements of static compliance, total resistance and airway resistance, and peak expiratory flow were evaluated before the technique, immediately after the technique and after aspiration. Two-way analysis of variance for repeated measures was used with Tukey's post hoc test, and p < 0.05 was considered significant.

**Results:**

Ventilator hyperinflation increased static compliance, which remained at the same level after aspiration (46.2 ± 14.8 *versus* 52.0 ± 14.9 *versus* 52.3 ± 16.0mL/cmH_2_O; p < 0.001). There was a transient increase in airway resistance (6.6 ± 3.6 *versus* 8.0 ± 5.5 *versus* 6.6 ± 3.5cmH_2_O/Ls^-1^; p < 0.001) and a transient reduction in peak expiratory flow (32.0 ± 16.0 *versus* 29.8 ± 14.8 *versus* 32.1 ± 15.3Lpm; p <0.05) immediately after the technique; these values returned to pretechnique levels after tracheal aspiration. There were no changes in the control condition, nor were hemodynamic alterations observed.

**Conclusion:**

Ventilator hyperinflation promoted increased compliance associated with a transient increase in airway resistance and peak expiratory flow, with reduction after aspiration.

## INTRODUCTION

Hospitalized patients exhibit excessive mucus production and impaired mucociliary clearance mechanisms.^(1)^ Some factors, such as prolonged recumbency, neurodegenerative conditions, advanced age and smoking, may inhibit mucus mobilization and elimination and/or reduce the effectiveness of coughing.^(2)^^)^ In addition, some diseases, such as pneumonia, bronchiectasis, chronic obstructive pulmonary disease, cystic fibrosis and asthma have the potential to increase the production of airway secretions.^(3)^ Thus, the accumulation of secretions in the airways results from a combination of the underlying disease and abnormal mucociliary activity due to changes in mucus production and composition, ciliary structure and function, and the cough mechanism.^(4)^

During mechanical ventilation, the primary determinants for the increase in mucus viscosity and changes in mucociliary clearance are changes in the mucociliary system, artificial airway, complications related to immobility, acquired muscle weakness with an impact on cough efficacy, and inadequate humidification.^(1,5)^ These factors are related to an increased risk of secretion retention and the development of atelectasis due to airway obstruction, which may cause impaired gas exchange, pulmonary infection and fibrosis, and progressive reduction of lung compliance.^(1,6)^

Lung hyperinflation can be performed manually (MHI), using a manual resuscitator or anesthesia bag, or by mechanical ventilation (i.e., ventilator hyperinflation - VHI). VHI was initially described by Berney and Denehy^(7)^ and consists of increasing the inspired volume through adjustments of the mode and/or parameters of the mechanical ventilator. It aims to mobilize and remove pulmonary secretions from the peripheral airways to mitigate the complications associated with their retention.^(8,9)^ Although there is no evidence of differences between MHI and VHI in terms of secretion clearance, respiratory mechanics or oxygenation,^(10)^ VHI has advantages related to the prevention of ventilator disconnection and the control of ventilatory variables.^(3)^ However, the effects of the VHI application method are worth mentioning. Studies have shown wide variability among ventilatory modes, and some results are unclear in terms of the precise adjustment of ventilatory parameters. Ventilatory adjustments have the potential to influence the movement of secretions and the gas distribution in the distal airways, which may affect the therapeutic effects.^(11,12)^

This is the first study to perform VHI in pressure-controlled ventilation (PCV) mode with individual adjustment of the inspiratory time according to the respiratory mechanics through a flow-time curve. The hypothesis of this study was that the adjustment of inspiratory time can optimize ventilation and increase the inspired volume, producing additional therapeutic effects on the displacement of secretions and on respiratory mechanics.

The objective of this study was to evaluate the effects of VHI on the mechanics of the respiratory system in mechanically ventilated patients.

## METHODS

A randomized crossover clinical trial with 38 mechanically ventilated adult patients was performed at the *Hospital Geral de Guarus* located in Campos dos Goytacazes, state of Rio de Janeiro (RJ), Brazil. The study was approved by the Research Ethics Committee of the *Institutos Superiores de Ensino do CENSA* (ISECENSA), and the legal guardians of the patients signed an informed consent form.

Patients older than 18 years who had pulmonary infection, had been mechanically ventilated for more than 96 hours under pressure-support ventilation (PSV) or PCV modes and had static compliance (C_st,rs_) between 25 and 70mL/cmH_2_O and positive end expiratory pressure (PEEP) between 5 and 8cmH_2_O were included in the study. Patients with hemodynamic instability confirmed by a mean arterial pressure lower than 70 mmHg or high doses of vasoactive amines, undrained pleural effusion or pneumothorax, intracranial hypertension, bronchospasm confirmed by pulmonary auscultation, adult respiratory distress syndrome (ARDS) or decompensated congestive heart failure were excluded from the study.

### Intervention

The order in which the interventions were performed was determined randomly; the interventions were performed at 6-hour intervals in 2 blocks of 20 patients. The order of the VHI and control (CTRL) conditions was determined by computer permutation, and the generated results were placed in envelopes numbered from 1 to 20, totaling 40 envelopes. The envelopes were opened sequentially at the time of data collection. The randomization process was hidden from the researcher.

For both interventions, the patients were placed in the dorsal decubitus position with the headboard raised 45º; they then underwent closed tracheal aspiration according to the recommendations of the American Association for Respiratory Care (AARC).^(13)^ In addition, the bacteriological filter of the ventilator was replaced, the cuff pressure was increased and the circuits of the mechanical ventilator were checked for leakages.

The VHI technique with the adjustment of inspiratory time was performed in the PCV mode. The inspiratory pressure was gradually increased every 5 cmH_2_O until a maximum pressure of 35cmH_2_O was reached according to patient tolerance, which was determined by the absence of coughing. PEEP remained unchanged throughout the study. After a maximum pressure of 35cmH_2_O (PCV + PEEP level) was reached, the inspiratory time was gradually increased until the inspiratory flow reached the baseline. Concurrently, the respiratory rate was decreased to allow the expiratory flow to also reach the baseline to avoid auto-PEEP. The technique was performed for 5 minutes and was followed by tracheal aspiration. For the CTRL condition, the patients were only positioned and aspirated, without changes in ventilatory parameters.

### Evaluation

Respiratory mechanics were evaluated before (PRE), immediately after the VHI or CTRL treatment (POST_imm_) and 10 minutes after aspiration (POST_asp_) using a Candle ventilator (Bird Products Corporation; Palm Springs, California, USA). The occlusion method was used at the end of inspiration in volume-controlled ventilation (VCV) mode, with a constant flow of 40L/minute and an inspiratory pause of 3 seconds. All patients were sedated (Ramsay 6). The ventilator screen was "frozen" to obtain the maximum pressures, P1 (the point separating the rapid and slow pressure drop immediately after the pause), plateau and PEEP, enabling the calculation of C_st,rs_, total resistance (R_rs_), airway resistance (R_aw_) and peak expiratory flow (PEF). Three consecutive measurements that were acceptable were taken at each time point, and the average of the two measurements with the lowest standard deviation was used. Each measurement was considered acceptable if no deflections were detected in the flow and pressure curves and/or there was no plateau along the inspiratory pause, which would suggest patient interference and the presence of leaks, respectively.

**Figure 1:**
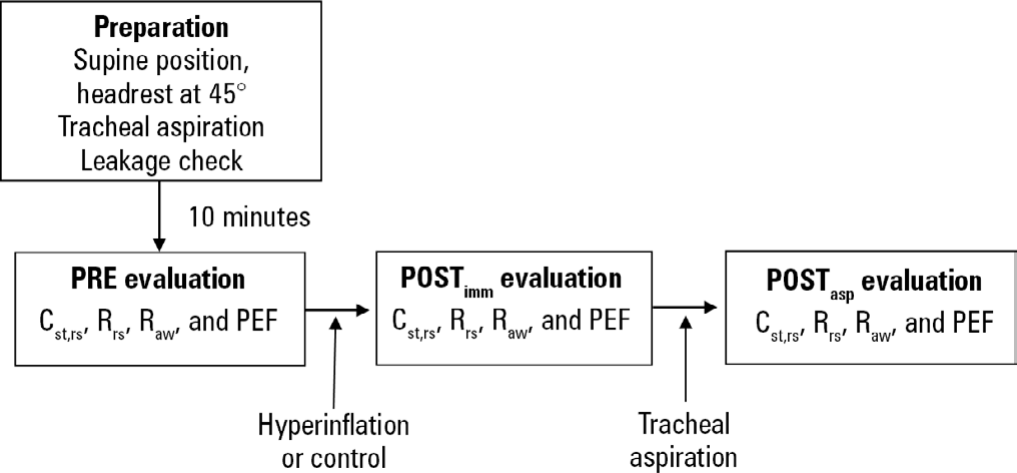
Study design. POST_imm_ - immediately after treatment; POST_asp_ - after aspiration; C_st, rs_ - static compliance; R_rs_ - total resistance; R_aw_ - airway resistance; PEF - peak expiratory flow.

Hemodynamics (heart rate and mean arterial pressure) and peripheral oxygen saturation were monitored throughout the protocol using a DIXTAL 2030 multiparameter monitor (Biomédica Ind. Com. LTDA; São Paulo, Brazil). The study design can be observed in figure 1.

### Statistical analysis

Mechanical measurements performed before and immediately after intervention and after aspiration were analyzed by two-way analysis of variance (ANOVA) for repeated measures with Tukey's post hoc test for results with normal distribution or with homogeneity of variances and were confirmed by the Shapiro-Wilk test and Levene's median, repectively. If the distribution was not normal, the Friedman test was used. A significance level of 5% was used. SigmaPlot^®^ 12.01 software (Systat Software Inc.; Richmond, California, USA) was used to analyze the results.

## RESULTS

A total of 38 patients were analyzed from August 2017 to March 2018. On the day of the study, all patients were under orotracheal intubation (OTI) or tracheostomy intubation and coupled to mechanical ventilation in the VCV or PCV mode. Table 1 shows the characteristics of the sample.

**Table 1:** Characteristics of the sample

Characteristics	
Age, years	59.4 (15.3)
Female sex	20 (52.6)
Ventilation time, days	14.3 (2.7)
PaO_2_/FiO_2_	301.9 (112.7)
Initial ventilatory mode, PSV	22 (57.9)
Diagnosis	
Stroke	17 (44.7)
Sepsis	8(21.0)
COPD	6 (15.7)
APE	2 (10)
CRF	2 (10)

PaO_2_ - partial pressure of oxygen; FiO_2_ - fraction of inspired oxygen; PSV - pressure support ventilation; COPD - chronic obstructive pulmonary disease; APE - acute pulmonary edema; CRF - chronic renal failure. The results are expressed as the mean (standard deviation) or n (%).

The time-adjusted VHI increased C_st,rs_ immediately after VHI compared with the pre-VHI value (p < 0.001) and remained constant after tracheal aspiration (p = 0.950). In the comparison between VHI and CTRL, C_st,rs_ was higher at POST_imm_ (47.8 ± 2.8 *versus* 43.2 ± 2.6mL/cmH_2_O, p < 0.001) and POST_asp_ (48.1 ± 2.9 *versus* 43.3 ± 2.5mL/cmH_2_O; p < 0.001), and there were no differences in PRE (42.4 ± 2.6 *versus* 42.6 ± 2.5mL/cm H_2_O_,_ p = 0.837). The values are shown in table 2.

**Table 2:** Respiratory mechanics

	PRE	POST_imm_	POST_asp_
C_st,rs_ (mL/cmH_2_O)			
VHI	46.2 ± 14.8	52.0 ± 14.9* (14.0%)	52.3 ± 16.0* (0.9%)
Control	46.8 ± 12.8	47.7 ± 13.4 (2.3%)	47.7 ± 12.7 (0.5%)
P-value	0.44	< 0.001	< 0.001
R_rs_ (cmH_2_O/L.s^-1^)			
VHI	15.0 ± 5.4	16.8 ± 6.2* (13.9%)	16.3 ± 6.3* (-2.4%)
Control	15.0 ± 5.6	15.3 ± 5.6 (2.8%)	14.9 ± 5.4 (1.7%)
P-value	0.97	0.02	0.04
R_aw_ (cmH_2_O/L.s^-1^)			
VHI	6.6 ± 3.6	8.0 ± 5.5* (24.1%)	6.6 ± 3.5† (-13.6%)
Control	6.8 ± 3.8	6.7 ± 3.9 (0.5%)	6.6 ± 3.8 (-0.1%)
P-value	0.25	0.01	0.48
PEF (Lpm)			
VHI	32.0 ± 16.0	29.8 ± 14.8* (-3.0%)	32.1 ± 15.3† (8.9%)
Control	32.9 ± 15.8	32.5 ± 16.1 (-0.2%)	32.4 ± 15.8 (0.6%)
P-value	0.24	0.21	0.16

C_st,rs_ - static compliance; VHI - hyperinflation with mechanical ventilator; R_rs_ - total resistance; R_aw_ - airway resistance; PEF - peak expiratory flow.

* Statistically significant differences compared to pre-VHI (PRE; p < 0.001);

† statistically significant differences compared to immediate post-VHI (POST_imm_; p < 0.001). Two-way analysis of variance for repeated measures with Tukey’s post hoc test. The results are expressed as the mean ± standard deviation.

The variables R_rs_, R_aw_ and PEF increased after VHI. R_rs_ increased immediately after VHI, without a statistically significant reduction after tracheal aspiration (p = 0.165). Comparing VHI and CTRL, R_rs_ was higher in the VHI in the POST_imm_ period (16.9 ± 1.0 *versus* 15.5 ± 0.9cmH_2_O/Ls^-1^; p = 0.021) and POST_asp_ period (16.3 ± 1.0 *versus* 15.1 ± 0.8cmH_2_O/Ls^-1^; p = 0.037), with no difference in the PRE (15.1 ± 0.8 *versus* 15.1 ± 0.9cmH_2_O/Ls^-1^; p = 0.974). R_aw_ increased immediately after VHI (p < 0.001), returning to baseline values after aspiration (6.5 ± 0.5 *versus* 7.7 ± 0.8 *versus* 6.5 ± 0.5cmH_2_O/Ls- ^1^; p < 0.001). Comparing VHI and CTRL, there was only a difference in the POST_imm_ period (p = 0.005); there were no differences in the PRE (p = 0.735) or POST_asp_ (p = 0.837). PEF decreased immediately after VHI (p = 0.034), returning to baseline values after aspiration (32.0 ± 16.0 *versus* 29.8 ± 14.8 *versus* 32.1 ± 15.3Lpm; p = 0.021). Comparing VHI and CTRL, there was only a significant difference in the POST_imm_ (p = 0.041); there were no differences in PRE (p = 0.243) or POST_asp_ (p = 0.350). No modifications were observed in R_rs_, R_aw_ or PEF in the CTRL condition.

During VHI, the inspired volume increased when the PCV mode was adjusted and after the time was adjusted (404.0 ± 85.2 *versus* 878.2 ± 219.2 *versus* 993.2 ± 345.2mL). The volumes increased by 117.7 ± 32.9% in PCV mode and by 144.6 ± 65.6% after the inspiratory time was adjusted. The inspiratory time obtained with VHI was 1.41 ± 0.35 seconds. No changes in heart rate, blood pressure or peripheral oxygen saturation were observed throughout the protocol in either group.

## DISCUSSION

The results of this study showed that VHI for 5 minutes in the PCV mode with increased inspiratory time promoted the displacement of secretions, confirmed by the behavior of the respiratory mechanics. The transient increase in total airway resistance, the slow pressure drop and the PEF suggest the displacement of pulmonary secretions of the peripheral airways to more central regions. The displacement of secretions promotes the expansion/recruitment of collapsed units and/or high time constants, with a consequent increase in C_st,rs_. This effect is due to increased collateral ventilation, elastic recoil pressure and expiratory flow, with a consequent increase in gas-liquid interaction.^(14)^ The reduction in R_aw_ and PEF after aspiration suggested that the secretions displaced to the more central airways were removed by aspiration.

Several authors have used C_st,rs_ as a clinical outcome to evaluate the therapeutic effects of MHI or VHI.^(7,14-19)^ Berney and Denehy^(7)^ performed a double crossover study comparing the MHI and VHI techniques, and VHI was performed in controlled volume mode, with a constant flow of 20Lpm and an inspiratory pause of 2 seconds. The protocol consisted of six series of six cycles, totaling 20 minutes, and the volume was increased by 200mL until a maximum pressure (Pmax) of 40cmH_2_O was reached. The authors observed that both techniques were equally effective for increasing C_st,rs_ and for the production of secretions, which increased by 11.6% after 30 minutes of VHI therapy and by 9.7% with MHI. In the present study, C_st,rs_ increased by 14.8% after 10 minutes of VHI with the optimization of inspiratory time. In another study comparing MHI and VHI, Dennis et al.^(20)^ performed a clinical trial with 46 patients with atelectasis or consolidation confirmed by X-ray. VHI was performed in synchronized intermittent mandatory ventilation (SIMV) with the VCV mode in four series of eight cycles. The volume was increased in 150-mL steps until a Pmax of 40cmH_2_O was reached or 250% of the tidal volume (TV) was obtained. However, the volume obtained during the interventions was not disclosed. The authors observed that VHI increased C_dyn,rs_ by 7.9%, but without differences between the techniques. In the present study, the inspiratory volume obtained was 244.6 ± 65.6% of the TV. Ahmed et al.^(21)^ compared the effects of MHI and VHI on 30 patients in the immediate postoperative period after mitral valve replacement. VHI was performed in the VCV mode with an increase in TV of 150%, a respiratory rate of 8 bpm, and a limited pressure of 35 mnHg (mean of 47.6cmH_2_O). C_dyn,rs_ increased by 6.6% after 1 minute of VHI; however, C_st,rs_ remained unchanged after 1 minute (5.6%) and after 20 minutes (4.8%). MHI did not modify C_st,rs_ and C_dyn,rs_. Savian et al.^(22)^ conducted a randomized crossover clinical trial with 14 patients in a general intensive care unit (ICU) comparing MHI and VHI associated with different PEEP levels (5, 7.5 and 10cmH_2_O). VHI was performed in the VCV mode for 3 minutes with TV at 130%, limited pressure of 40cmH_2_O and respiratory rate adjusted between 7 and 8 pm, followed by tracheal aspiration. Both techniques also increased C_st,rs_, but the results were only significant 30 minutes after VHI; an increase of 12% (6.0 mL/cmH_2_O) was observed, with no differences between PEEP levels. In the present study, there was an increase of 6.0 ± 9.9mL/cmH_2_O. Anderson et al.^(10)^ performed a systematic review comparing the effects of MHI and VHI on critically ill patients. Only these four previously cited clinical trials were selected for the analysis of the results. Assmann et al.^(23)^ performed a randomized crossover trial of 50 patients who received aspiration alone or VHI in VCV mode at 150% of the ideal tidal volume or in PCV mode with increments of 10cmH_2_O, and the maximum pressure was limited to 40cmH_2_O. VHI increased dynamic compliance and expired tidal volume, in addition to mobilizing a greater volume of secretions.

Few studies have performed VHI in pressure-controlled modes (PCV or PSV). Most clinical studies performed VHI under controlled volume ventilation, with progressive volume increases between 150^(20)^ and 200mL^(15)^ or by means of fixed proportional increases of 130%^(23)^ or 150%^(21,23)^ of TV. Lemes et al.^(14)^ performed VHI in PSV mode with a total pressure increase up to 40cmH_2_O; PEEP was maintained, and the patients were positioned in lateral decubitus. The authors observed a 13.8% increase in C_st,rs_. Another study associated MHI with Flutter^®^-valve coupling to the expiratory port of the mechanical ventilator in a randomized crossover clinical trial. The authors used the PCV mode, with inspiratory pressure adjustment at 25cmH_2_O associated with Flutter-produced PEEP (approximately 14.7cmH_2_O)^(16)^ and a total maximum pressure of 40cmH_2_O. Silva et al.^(24)^ performed manually assisted cough with inspiratory pressure adjustment at 20cmH_2_O above the PEEP of 15cmH_2_O, for a total pressure of 35 cmH_2_O.

Dennis et al.^(25)^ analyzed the profiles of 64 Australian tertiary ICUs and found that 39% of the interviewees used VHI. Most respondents limited therapy by peak pressure and achieved an average of 37cmH_2_O, ranging from 25 to 40cmH_2_O. Physiotherapists using the VCV mode increased the inspiratory volume between 15 and 200%. In the ICUs that performed VHI using pressure-controlled modes, the pressure was increased stepwise from 2 to 5cmH_2_O. The application time ranged from 5 to 30 minutes. In the present study, increments of 5cmH_2_O were applied until a maximum pressure of 35cmH_2_O was reached for 5 minutes.

The effectiveness of bronchial hygiene is also related to inspiratory volume, elastic recoil pressure, pattern of secretion distribution and higher time constants. The increase in inspiratory time was associated with VHI, with the objective of increasing the inspiratory volume and distribution of ventilation in the alveolar units with high time constants. Patients with obstructive patterns seem to benefit from the increase in inspiratory time because they have "slow" flow patterns.^(26)^ In addition, flow values close to the end of inspiration are associated with the terminal or peripheral airways.^(27)^ In some patients, increased inspiratory time promoted the cough reflex. When the pressure increased, the inspiratory volume increased 117.7 ± 32.9%, but the added inspiratory time increased the TV by 144.6 ± 65.6%. Silva et al.,^(24)^ when performing manually assisted cough with VHI, increased the inspiratory time to 2 seconds for all patients, regardless of the respiratory mechanics. Berney and Denehy^(7)^ applied slow inspiratory flow (20Lpm) and 200mL increments in the TV until the maximum pressure reached 40cmH_2_O, with a consequent increase in inspiratory time. In the present study, the inspiratory time was individually adjusted according to the inspiratory and expiratory flow curves so that both reached the baseline. Thus, low flow values occurred at the end of inspiration.

During mechanical ventilation, the ventilatory mode, the parameters used and the mechanical properties of the respiratory system may influence the mobilization of secretions and may result in the accumulation of secretions in the peripheral airways.^(11)^ For VHI or MHI to mobilize secretions, the inspiratory and/or expiratory flow rates must be modulated to favor the movement of secretions to the proximal airways. Thomas^(28)^ suggested three flow characteristics that may contribute to bronchial hygiene: (1) peak inspiratory flow (PIF) less than 90% of PEF; (2) PEF > 40L/minute; and (3) difference between PEF and PIF (*bias flow*) of at least 17 L/minute. The author analyzed the PCV mode using different combinations of inspiratory pressure adjustments (20, 30, 35 and 40cmH_2_O), PEEP (0, 5, 10 and 15cmH_2_O) and inspiratory time (1, 2 and 3 seconds). The increase in inspiratory time was the variable that promoted the largest number of ventilatory combinations that met criteria 1 and 3.

Studies have shown that the MHI and VHI techniques promote the improvement of ventilatory parameters and respiratory mechanics without promoting hemodynamic changes. Measurements such as mean arterial pressure, heart rate and peripheral oxygen saturation (SpO_2_) are used to measure the hemodynamic impact of respiratory physiotherapy in the ICU.^(14,18)^ During the techniques performed in the present study, no significant changes in blood pressure, heart rate or peripheral oxygen saturation were observed.

The main limitations of the present study were the absence of secretion volume measurement and blinding. Further studies are needed to evaluate the effects of technique optimization by increasing the inspiratory time and to determine the effects under specific disease conditions. Moreover, the measurement of the variables only 10 minutes after tracheal aspiration did not allow a determination of the duration of changes in the respiratory mechanics.

## CONCLUSION

Hyperinflation with mechanical ventilator promoted increased compliance associated with a transient increase in airway resistance and peak expiratory flow that decreased after aspiration.
